# Retroperitoneal Lipoma Presenting with Nutcracker-Like Phenomenon

**DOI:** 10.1155/2013/893242

**Published:** 2013-11-18

**Authors:** Seiichi Saito

**Affiliations:** Art Park Urology Hospital & Clinic, Ishiyama-Higashi 3-1-31, Sapporo 005-0850, Japan

## Abstract

Retroperitoneal lipoma presenting with a nutcracker-like phenomenon is extremely rare. I experienced a case of a 65-year-old man presenting with left flank pain and macrohematuria intermittently for 3 years. Computed tomography revealed a lipoma at the left pedicle of the kidney, 30 mm in diameter, causing a curving of the left renal artery and dilatation of the left renal vein. This patient was treated successfully by retroperitoneoscopic resection of the lipoma. There have been no symptoms for 10 years after the operation.

## 1. Introduction

The nutcracker syndrome was first reported in 1972 by Schepper [1]. Compression of the left renal vein between the superior mesenteric artery and aorta can cause venous hypertension and formation of ureteral and renal pelvic venous varicosities. Related symptoms and hematuria have been described as the nutcracker syndrome previously [1, 2], but it remains unclear why compression of the left renal vein by the superior mesenteric artery afflicts only a few patients and which anatomical details are related to the pathophysiological mechanism. Actually, there are many other causes of compression of the left renal vein such as aneurysm, curving of the left renal artery, or renal arterial and venous anomalies [3–6]. I herein report a case with retroperitoneal lipoma presenting with a nutcracker-like phenomenon.

## 2. Case Report

**Figure 1:**
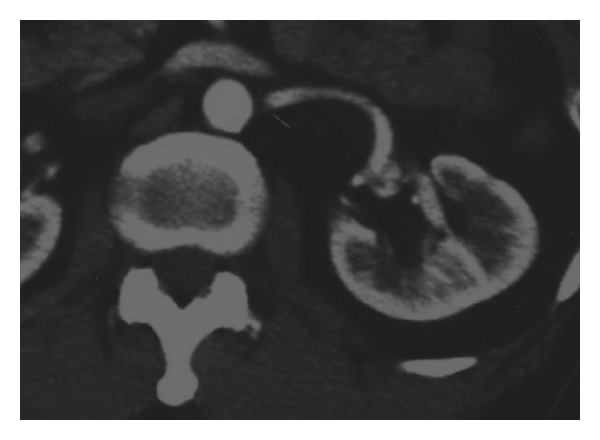
CT scan reveals retroperitoneal lipoma around the left renal pedicle, 30 mm in diameter, causing a curving of the left renal artery and dilatation of the left renal vein.

**Figure 2:**
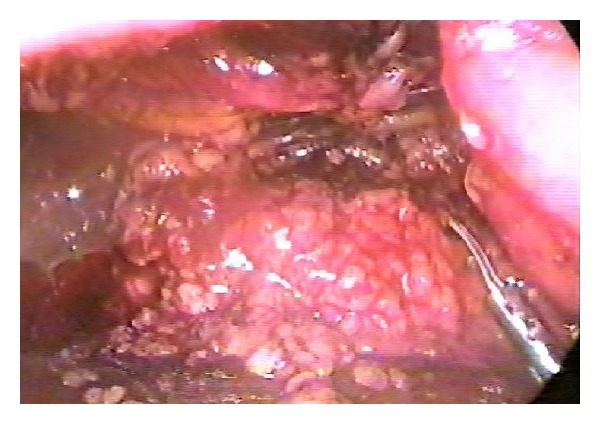
Retroperitoneal lipoma is a yellow lobulated and encapsulated mass under laparoscopy.

**Figure 3:**
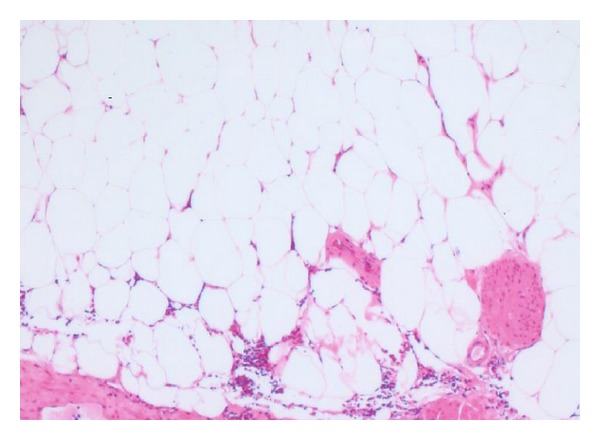
Histopathologically, retroperitoneal lipoma consists entirely of mature fat.

A 65-year-old man has been presenting with intermittent gross hematuria and left flank pain for 3 years. There was no history of renal trauma, urinary tuberculosis, urinary bilharziasis, or other diseases. Physical examination revealed no abnormal findings. Laboratory test results were within normal ranges except for microscopic hematuria. Ultrasonography of the urinary tract and excretory urography were normal. Computerized tomography confirmed retroperitoneal lipoma around the left renal pedicle, 30 mm in diameter, causing a curving of the left renal artery and dilatation of the left renal vein (Figure 1). His condition was diagnosed as retroperitoneal lipoma that caused abnormal renal arterial traveling and compression of the renal vein. On June 25, 2003, retroperitoneoscopic lipoma resection was performed with the standard 3-port technique. Grossly, lipoma is a yellow lobulated and encapsulated mass (Figure 2); histopathologically, it consists entirely of mature fat (Figure 3).

Operation time was 60 minutes without major bleeding. No further episodes of gross hematuria or left flank pain have occurred for 10 years after the operation.

## 3. Discussion

The nutcracker syndrome is an unusual cause of benign lateralizing hematuria, which is caused by the high pressure on the left renal vein and is difficult to treat [1, 2]. Surgical techniques include left renal autotransplantation, reanastomosis of the left renal vein to the inferior vena cava, and intravenous stent placement [2, 7]. The indication for an operation for the nutcracker syndrome is severe, persistent, or recurrent hematuria causing anemia, as observed in most of patients, and abdominal or flank pain probably caused by ureteral passage of blood clots. Patients with microscopic hematuria without anemia may be followed closely without further treatment. This patient hoped for minimally invasive surgery to relieve this phenomenon. As a result, this case was treated successfully by retroperitoneoscopic resection of the lipoma. Retroperitoneoscopic surgery is desirable for selected patients because it is the most minimally invasive. Recently most of kidney surgery is performed by the retroperitoneoscopic approach. This approach has potential advantages, since the kidney is a retroperitoneal organ, including early control of the renal vessels and nonviolation of the peritoneal cavity, with resultant minimal ileus and rapid recovery. Following balloon dilation and placement of 3 ports, retroperitoneal space is created easily and safely [8].

Although retroperitoneal lipoma is not rare, a case presenting with nutcracker-like phenomenon has not hitherto been reported to the best of my knowledge. I should always think about various possibilities for every patient presenting with nutcracker syndrome.
